# Mammitis and Mammary Abscesses Treated by Bandaging

**Published:** 1876-02

**Authors:** L. A. Dugas

**Affiliations:** Professor of Surgery in the Medical College of Georgia


### MAMMITIS AND MAMMARY ABSCESSES TREATED BY BANDAGING.

By L. A. DUGAS, M.D., LL.D., Professor of Surgery in the Medical College of Georgia.

*Read before the Medical Association of Georgia, in April,* 1875.

There is nothing more trying to a woman than the supervention of inflammation of the breasts after parturition, and I know of no disease more injudiciously managed by nurses, midwives, and sometimes by physicians. All this mal-practice seems to me to depend upon an erroneous appreciation of the nature of the disease and of the effects of the remedial agents employed.

What is it with which we have to contend? The sage dame will reply, that the milk is accumulating faster than the infant can remove it, and that unless it be drawn off it will coagulate and form "cakes" in the breast. In order to avoid this caking, therefore, the mother is tortured with the frequent application of her infant to the nipple; the children of the neighborhood, the puppies, kittens, pumps of various kinds, and perhaps the mouth of the nurse herself, will be brought into requisition to accomplish the important purpose. But if, notwithstanding all these efforts, "caking" of the breast takes place, it must be "softened" by frictions with various liniments more or less irritating, by warm fomentations, hot and ponderous poultices, etc. The result of such treatment can easily be predicted, for it is difficult to imagine a plan better calculated to produce suppuration more or less extensively in the affected organ.

What, then, is the true nature of the affection? In order to understand this we should bear in mind that the structure of the breast is partly erectile, and that it may become the seat of a great afflux of blood under certain circumstances. Before child-bearing this afflux is limited to the nipple and its areola, and readily subsides after the occasional excitation has ceased. But when a woman becomes a mother, the great determination of blood concentrated upon the uterus for the sustentation of the child during the preceding nine months, is now directed for the same purpose towards other organs. The offspring is now to be provided for at the great and marvelous founts represented by the mammæ; and this transition is more or less trying, especially when it occurs for the first time, and in a constitution enfeebled by the sedentary habits imposed upon women by the so-called refinements of high life. The determination of blood is then very great; perhaps greater than demanded by the requirements of the infant; and this congestion may run into positive inflammation and suppuration if not moderated. It is from this blood that the milk is secreted, and, when secreted, the milk remains in the milk tubes until it is drawn off by the suction power of the infant's mouth. Whenever the child is applied to the breast, he not only draws what is in the milk tubes, but his suction provokes an afflux of blood to the breast and an increased secretion of milk so as to supply his wants. This flowing of blood into the breast is distinctly felt by the mother whenever she nurses the child, and is attributed by her to the flowing of milk. Now, milk does not *flow* into the breast; it is *formed* there. It should then be distinctly recollected that the child's suction is always attended with an increased afflux of blood to the organ. Any irritation or friction of the breast will produce the same result, and so will hot poultices and fomentations.

I have heard physicians urge in favor of multiplying the means of drawing off the milk, that, inasmuch as the milk is separated or derived from the blood, the more milk the organ secretes the less blood it will contain; or, in other words, that the circulation will be diminished in a direct ratio with the secretion of milk. This is a fundamental error, at variance with all we know of physiology. The more active the secretion of a gland the greater must be the demand for blood. One column of blood having yielded such of its elements as may be fitted for the secretion, it is immediately replaced by another containing new materials, and we have thus a succession of columns more or less rapidly supplied according to the demand of the gland. Increased secretion must necessarily imply increased activity in the circulation, or an increased afflux of blood. In pathology we look upon the return of secretion as one of the first indications of a subsidence of congestion or of inflammation, and not as the *cause* of its diminution.

From these premises it must be manifest that if we wish to lessen the determination of blood to the mamma, or to prevent it from causing inflammation, we should not only avoid what may increase it, but also use such measures as may tend to lessen it. Instead, therefore, of frequent applications of the child to the breast, he should, on the contrary, be suckled as seldom as possible. Instead of frictions and warm applications, we should make use of such gentle compression as will force out the blood from its distended vessels, and keep the organ as cool as possible by avoiding unnecessary clothing. Quinine should be given if there be any fever. In this way we may expect very generally to succeed in averting the threatened affection.

I have for many years entertained these views of the pathology and treatment of mammitis, and experience has only confirmed me of their correctness. I was, however, for a long time perplexed by the difficulty of making such compression as might be effectual, of easy application, and would offer no impediment to nursing the child. I at first used the roller and other mammary bandages recommended in the books, but found them all difficult, cumbersome, and ineffectual from the facility with which they became displaced. I then tried adhesive plaster spread upon thin sheep-skin, and subsequently a coating of collodion. The adhesive plaster is with difficulty obtained of good quality, is troublesome to take off and readjust daily, as it should be, and is too warm in summer. The collodion is also rarely to be procured of good quality, and is apt to induce excoriations about the margins of the coating. I have consequently long since abandoned all these expedients, and devised a bandage which combines all the requisites. It consists simply of a bit of cotton or linen shirting, about ten inches wide and long enough to surround the thorax and to be secured in front by digitations similar to other "many-tailed" bandages. This is to be applied from the axilla down, pass around the chest and over the mamma, and be tied in front of the sternum. It effectually compresses both organs, and may be removed, loosened or tightened, according to the exigences of the case and without any difficulty whatever. If only one breast is affected, the bandage may be so split as not to cover the whole of the other; but there is no harm done by compressing both. The child may be nursed through an aperture made in the bandage for the nipple. If the nipple or its surroundings be so implicated as to require compression also, the bandage may have to be removed whenever the child is nursed, if splitting will not answer the purpose.

As soon as the congestion becomes painful or threatening, the bandage should be applied with such moderate tightness as will relieve pain, and continued until the trouble has entirely subsided. If applied too tightly it may suspend the secretion of milk so effectually as to necessitate the assiduous application of the child, or a resort to the pump, in order to re-establish the supply of milk. This use of the bandage should not prevent the administration of quinine whenever fever occurs.

When suppuration takes place, the pus should be let out by the free use of the bistoury, and a linen tent renewed daily. There should also be an opening in the bandage opposite the incision, for the escape of pus. The relief afforded by the bandage is so complete that the pus may sometimes increase and make its way to the surface without being detected. The physician should therefore be on the lookout for such an occurrence, especially in cases in which the application of the bandage has been too long delayed.

Abscesses may occur beneath the areola, between the skin and gland, within the glandular structure, and behind the gland. The last are the most formidable and difficult to control; yet they can always be mastered by the bandage. This bandage is adapted to every stage of mammary inflammation. If too late to prevent suppuration, it will lessen the size of the abscess, and, after an opening has been made for the escape of pus, it will bring together the walls of the abscess and speedily effect a cure. One of its greatest advantages is that it relieves the patient of all pain as soon as it is applied, by removing the distention to which the unsupported tissues are subjected by the congestion, the tumefaction, or the accumulation of pus. It also effectually prevents the multiplication of abscesses so frequently attending the ordinary methods of treatment, and which prolong the sufferings of the woman indefinitely.

